# Effect of Different Dilution Methods and Ratios of Ram Semen on Sperm Parameters after Cryopreservation

**DOI:** 10.3390/ani14060907

**Published:** 2024-03-15

**Authors:** Liuming Zhang, Xuyang Wang, Caiyu Jiang, Tariq Sohail, Yuxuan Sun, Xiaomei Sun, Jian Wang, Yongjun Li

**Affiliations:** Key Laboratory for Animal Genetics & Molecular Breeding of Jiangsu Province, College of Animal Science and Technology, Yangzhou University, Yangzhou 225009, China; 18352767281@163.com (L.Z.); 18705271593@163.com (X.W.); m19352674787@163.com (C.J.); drtariqsohail34@yahoo.com (T.S.); 19802622001@163.com (Y.S.); xiaomeisun_yz@163.com (X.S.); jianwang1223@163.com (J.W.)

**Keywords:** dilution rates, *Hu* ram, cryopreservation, spermatozoa motility, spermatozoa functional integrity, spermatozoa ROS

## Abstract

**Simple Summary:**

The appropriate dilution rate is crucial for the spermatozoa’s survival. Both excessively large and excessively small dilution rates will diminish the effectiveness of semen preservation. However, the dilution method and ratio for preserving *Hu* ram semen through cryopreservation are currently unclear. Therefore, the study aimed to analyze the effects of various dilution methods and ratios on the spermatozoa motility parameters and functional integrity of *Hu* ram semen after cryopreservation. The results showed that employing the correct dilution method and ratio (two-step dilution 1:3, 1:2) could improve the preservation effect of semen.

**Abstract:**

The dilution method and ratio were tested to assess their effects on the *Hu* ram semen after cryopreservation. Experiment I aimed to explore the effect of various dilution ratios (1:1, 1:2, 1:3, 1:4) of diluent I (Tris-based and egg yolk) under the condition of 1:1 dilution of diluent II (diluent I and glycerol) on the *Hu* ram semen preserved in liquid nitrogen regarding spermatozoa motility and kinetic parameters. Experiment II aimed to investigate the effect of various dilution ratios (1:1, 1:2, 1:3, 1:4) of diluent I under the condition of 1:2 dilution of diluent II to the *Hu* ram semen for cryopreservation on spermatozoa motility and kinetic parameters. The purpose of experiment III is to assess the effect of various dilution methods and ratios on the cryopreservation of *Hu* ram semen by detecting spermatozoa motility, kinetic parameters, plasma membrane integrity, acrosome integrity and reactive oxygen species (ROS) level. Experiment III includes four groups: one-step dilution method and two-step dilution method. The two-step dilution method includes two groups: 1:2, 1:1 and 1:3, 1:2, and the one-step dilution method includes two groups: 1:5 and 1:11. The results indicated that the post-thawed spermatozoa total motility (TM), progressive motility (PM) and average motion degree (MAD) were highest in the 1:2 group and significantly higher (*p* < 0.05) than those in the 1:1 and 1:4 groups under the condition of 1:1 dilution of diluent II. The post-thawed spermatozoa TM and PM of the 1:3 group were significantly higher (*p* < 0.05) than those of the other groups under the condition of 1:2 dilution of diluent II. The post-thawed spermatozoa TM, PM, plasma membrane integrity and acrosome integrity of the two-step group (1:3, 1:2) were the highest and significantly higher (*p* < 0.05) than those in the other groups. Additionally, the post-thawed spermatozoa ROS level of the two-step group (1:3, 1:2) was significantly lower (*p* < 0.05) than that in the one-step groups (1:5 and 1:11). Therefore, a two-step dilution (1:3, 1:2) was found to be the most suitable method and ratio for diluting the *Hu* ram semen after cryopreservation.

## 1. Introduction

Artificial insemination (AI) refers to the method of collecting semen artificially and transferring the processed semen to the reproductive organs of female animals in estrus [1]. AI is an advanced method of mating. Compared to natural mating, AI has made significant advancements and is an important technical method for the advancement of modern animal husbandry [2]. AI technology offers several advantages, including improving the utilization rate of superior male animals, increasing the conception rate of female animals, preventing the spread of certain reproductive tract diseases, addressing challenges related to crossbreeding and mating, overcoming regional limitations in female breeding and facilitating embryo transfer, synchronous estrus and other reproductive techniques [3,4,5]. In addition to the influence of female body condition, estrus detection and insemination methods, the efficiency of AI is also closely related to semen quality. Several factors affect semen quality, including semen preservation methods, dilution components, antibiotics, semen collection frequency and dilution times [6,7].

The concentration of spermatozoa is crucial for the survival time of spermatozoa. Appropriate dilution rates can increase the volume of semen, enhance the utilization rate of spermatozoa, and improve the effectiveness of multiple AI procedures [8,9]. During the preservation of semen, the metabolism of spermatozoa continuously consumes nutrients and produces metabolic wastes and oxidative factors, which can affect the effectiveness of semen preservation. Therefore, when the spermatozoa concentration is too high, the spermatozoa density exceeds the capacity of the diluent to provide sufficient nutrients for sperm movement, and the buffer and antioxidant substances in the diluent are unable to maintain pH stability and redox balance [10]. When the concentration of spermatozoa is too low, the level of beneficial substances in seminal plasma decreases. This reduction diminishes the protective ability of spermatozoa, resulting in a dilution effect and a shortened effective preservation time of semen [11]. Excessive dilution rate can lead to a decrease in spermatozoa density and the number of effective spermatozoa while increasing the insemination volume, and due to the unique structure of the sheep’s reproductive tract, the increase in semen volume can exacerbate the reflux of spermatozoa from the cervix, ultimately reducing the pregnancy rate [12,13]. Therefore, it is crucial to find the appropriate dilution method and ratio for semen preservation and AI.

The *Hu* sheep is a unique breed of sheep in China, known for its early sexual maturity, rapid growth and development, high fecundity, suitability for indoor rearing, strong adaptability to various environments, and excellent meat production performance [14]. China has a large population and limited land, leading to a growing demand for mutton [15]. As a result, indoor sheep farming has become the predominant method of breeding, and the *Hu* sheep breed aligns with this current development trend. Currently, the breeding scale of *Hu* sheep in China is expanding, but there is still a shortage, especially in meeting the market demand for meat products and lambskin processing. In order to promote the sustained and rapid development of the *Hu* sheep breeding industry, it is essential to utilize AI and other related reproduction technologies.

In the study of frozen semen in sheep, the freezing methods such as dilution method and ratios are different. Chen [16] adopted a one-step dilution method of 7.5 times, and the post-thawed Mongolian sheep’s spermatozoa progressive motility (PM) reached 39%. He [17] used a two-step dilution method of 5 times, and the post-thawed Dorset rams’ spermatozoa total motility (TM) reached 31%. Fang [18] used a one-step dilution method of 2.5 times, and the post-thawed small-tailed Han sheep’s spermatozoa TM reached 13%. However, the dilution method and ratios of *Hu* ram semen in cryopreservation are not clear. Therefore, the purpose of this study is to provide a reference for improving the preservation technology of *Hu* ram semen in production practice by examining spermatozoa TM, PM, kinematic parameters, membrane integrity, acrosome integrity and reactive oxygen species (ROS) level.

## 2. Materials and Methods

### 2.1. Experimental Design

Experiment 1: The study investigated the impact of different dilution ratios of diluent I (1:1, 1:2, 1:3, 1:4) under the condition of 1:1 dilution of diluent II on the motility and kinetic parameters of *Hu* ram semen preserved in liquid nitrogen (−196 °C). TM, PM, straight-line velocity (VSL, μm/s), curvilinear velocity (VCL, μm/s), average path velocity (VAP, μm/s), amplitude of lateral head displacement (ALH, μm) and average motion degree (MAD, °/s) of the four groups were evaluated.

Experiment 2: The study aimed to investigate the impact of different dilution ratios of diluent I (1:1, 1:2, 1:3, 1:4) under the condition of 1:2 dilution of diluent II on spermatozoa motility and kinetic parameters after cryopreservation. TM, PM, VSL, VCL, VAP, ALH and MAD of the four groups were evaluated.

Experiment 3 aimed to investigate the one-step and two-step dilution methods. The two-step dilution method included two groups: 1:2, 1:1 and 1:3, 1:2, and the one-step dilution method included two groups: 1:5 and 1:11. The purpose of this experiment was to assess the effect of various dilution methods and ratios on the cryopreservation of *Hu* ram semen by measuring spermatozoa motility (TM and PM), kinetic parameters (VSL, VCL, VAP, ALH and MAD), plasma membrane integrity, acrosome integrity and ROS level.

### 2.2. Semen Collection and Diluent Preparation

The procedures for animals during the experiment complied with the regulations of the Animal Ethics Committee of Yangzhou University (Approval ID: 202206132). The study utilized five mature and healthy *Hu* rams, which were housed at the sheep facility within Yangzhou University (Wenhuilu campus, Yangzhou, China, 119.429731°, 32.395371°). The rams aged 2~3 years were fed 0.7 kg concentrate/day and straw–hay mixtures during the study period following fertility examination. Furthermore, water and mineral blocks were provided freely to the rams. *Hu* sheep were known to be in estrus all year round. From April to June 2023, ram semen was collected three times a week (a total number of 75 ejaculates) using an artificial vagina. After semen collection, all samples were maintained at 37 °C and transported to the laboratory within 30 min for semen sample quality evaluation, including assessment of volume (0.5 to 1.5 mL), concentration (≥2.5 × 10^9^ spermatozoa/mL) and total motility (≥80%). The qualified semen samples were pooled together for subsequent experiments.

The basic diluent was Tris-based, composed of 3.64 g Tris, 1.82 g citric acid, 0.5 g glucose, 20,000 IU sodium penicillin and streptomycin sulfate for 100 mL of distilled water. The freezing diluent I contained 80% (*v*/*v*) basic diluent and 20% (*v*/*v*) egg yolk. The freezing diluent II contained 94% (*v*/*v*) freezing diluent I and 6% (*v*/*v*) glycerol.

### 2.3. Semen Cryopreservation and Post Thaw

The semen freezing protocol of *Hu* rams was based on the research results of Wu [14] and improved upon this foundation. In experiment 1, semen samples were diluted at the ratios of 1:1, 1:2, 1:3 and 1:4, respectively, using freezing diluent I. Afterward, the samples were wrapped in a towel and stored at 4 °C for 2.5 h. Subsequently, isothermal freezing diluent II was added in a 1:1 ratio, and the samples were kept at 4 °C for 2.5 h. In experiment 2, semen samples were diluted at ratios of 1:1, 1:2, 1:3 and 1:4, respectively, using freezing diluent I. The samples were then wrapped in a towel and kept at 4 °C for 2.5 h. Subsequently, isothermal freezing diluent II was added at a ratio of 1:2, and the samples were kept at 4 °C for 2.5 h. In experiment 3, for the two-step dilution method, the semen samples were initially diluted at ratios of 1:2 and 1:3, respectively, using freezing diluent I. Afterward, the samples were wrapped in a towel and kept at 4 °C for 2.5 h. Subsequently, isothermal freezing diluent II was added in ratios of 1:1 and 1:2, respectively, and the samples were kept at 4 °C for 2.5 h. For the one-step dilution method, semen samples were diluted at ratios of 1:5 and 1:11, respectively, using freezing diluent II. Afterward, the samples were wrapped in a towel and kept at 4 °C for 5 h. Subsequently, all semen samples were promptly transferred into 0.25 mL straws at 4 °C. All the straws were held 2 cm above the liquid nitrogen for 20 min and then immediately plunged into the liquid nitrogen.

After being stored in liquid nitrogen for at least 1 week, four straws from each group were randomly selected, thawed at 70 °C for 5 s, and then utilized for further investigation and analysis.

### 2.4. Spermatozoa Motility and Kinetic Parameters Assessment

The spermatozoa motility (TM and PM) and kinetic parameters (VSL, VCL, VAP, ALH and MAD) were analyzed using a computer-assisted spermatozoa analyzer (CASA, ML-608JZ II Mailang, Nanning, China) equipped with a warm stage. Samples were diluted to 3.85 × 10^7^ spermatozoa/mL using a Tris-based diluent and then incubated in a water bath at 37 °C for 3 min. A total of 1.4 µL of semen was placed on a MACRO spermatozoa counting chamber (YA-1, Yucheng, Nanjing, China), and a phase-contrast microscope (ML-800, Mailang, Nanning, China) equipped with a CCD-camera (MD06200C, Mailang, Nanning, China) was used for the assessment. And the CASA software (ML-608JZ II) recorded data at 30 frames per second.

### 2.5. Spermatozoa Membrane Integrity Assessment

The spermatozoa membrane integrity was analyzed using the hypo-osmotic swelling test (HOST). Briefly, the samples were diluted to a concentration of 2 × 10^8^ spermatozoa/mL using a Tris-based diluent and 20 µL of diluted semen sample was added to 200 µL of hypotonic solution (0.245 g of sodium citrate and 0.45 g of fructose dissolved in 50 mL of water). The samples were incubated in a 37 °C water bath for 30 min. After incubation, 1.8 µL of the mixture was spread onto a ruby spermatozoa counting plate and examined under a microscope (CX31, Olympus, Tokyo, Japan) at a magnification of 400×. A total of 200 spermatozoa per group were assessed. Spermatozoa with a curled tail were considered to have intact plasma membrane.

### 2.6. Spermatozoa Acrosome Integrity Assessment

The spermatozoa acrosome integrity was assessed using FITC-PNA combined with PI fluorescence staining. Briefly, the samples were diluted to 3.21 × 10^8^ spermatozoa/mL using a Tris-based diluent, and 100 µL diluted samples were mixed with 2 µL of FITC-PNA (200 µg/mL) and 2 µL of PI (0.5 mg/mL). The samples were incubated at 37 °C for 10 min in the dark and then mixed by adding 700 µL of PBS. They were immediately assayed for spermatozoa acrosome integrity using flow cytometry (Beckman Coulter, Shanghai, China). Flow cytometry was configured to detect 10,000 spermatozoa, and those exhibiting FITC^−^/PI^+^ and FITC^−^/PI^−^ were counted as spermatozoa with intact acrosomes.

### 2.7. Spermatozoa ROS Level Assessment

The spermatozoa ROS level was detected using the DCFH-DC probe. Briefly, the samples were diluted to 3.21 × 10^8^ spermatozoa/mL using a Tris-based diluent, and 50 µL of diluted semen was mixed with 2 µL of DCFH-DC (10 mM). We incubated the sample at 37 °C in the dark for 30 min, then added PBS to wash the sample. The sample was then resuspended in 400 μL of PBS, and the fluorescence intensity was measured using a multifunctional microplate reader with excitation at 488 nm and emission at 525 nm. The level of ROS was indicated by fluorescence intensity.

### 2.8. Statistical Analysis

All data were analyzed using Statistical Product and Service Solutions (SPSS 25.0 for windows; SPSS Inc., Chicago, IL, USA). The Shapiro–Wilk normality analysis was performed to detect whether the data conform to the normal distribution. The data showed normal distribution, and the Duncan test by one-way ANOVA tests was performed to assess the difference in these parameters. All results were expressed as the Mean ± SEM, and a *p* value of <0.05 (*p* < 0.05) was considered significant. All experiments were performed with five replicates. 

## 3. Results

### 3.1. Effect of Different Dilution Ratios of Diluent I under the Condition of 1:1 Dilution of Diluent II on Spermatozoa Motility and Kinetic Parameters after Cryopreservation

As shown in Table 1, the post-thawed spermatozoa TM, PM and MAD of the 1:2 group were the highest and significantly greater (*p* < 0.05) than those of the 1:1 and 1:4 groups. However, they were not significantly higher (*p* > 0.05) than those of the 1:3 group. The post-thawed spermatozoa VSL of the 1:2 group was the highest and significantly higher (*p* < 0.05) than that of the 1:1 group. However, it was not significantly higher (*p* > 0.05) than the 1:3 and 1:4 groups. Additionally, these groups did not show significant (*p* > 0.05) differences from each other in terms of spermatozoa VCL, VAP and ALH.

### 3.2. Effect of Different Dilution Ratios of Diluent I under the Condition of 1:2 Dilution of Diluent II on Spermatozoa Motility and Kinetic Parameters after Cryopreservation

The post-thawed spermatozoa TM and PM of the 1:3 group were significantly higher (*p* < 0.05) than those of the other groups, as shown in Table 2. The post-thawed spermatozoa VCL, VAP and ALH of the 1:3 group were the highest and significantly higher (*p* < 0.05) than those of the 1:2 group, but they were not significantly higher (*p* > 0.05) than the 1:1 and 1:4 groups. The post-thawed spermatozoa MAD of the 1:3 group was significantly higher (*p* < 0.05) than that of the 1:1 group, but it was not significantly higher (*p* > 0.05) than that of the 1:2 and 1:4 groups. There was no significant difference (*p* > 0.05) in spermatozoa VSL between the groups.

### 3.3. Effect of Different Dilution Methods and Ratios of Diluent on Spermatozoa Motility and Kinetic Parameters after Cryopreservation

As shown in Table 3, the post-thawed spermatozoa TM and PM of the two-step group (1:3, 1:2) were the highest and significantly higher (*p* < 0.05) than those of the other groups. The post-thawed spermatozoa VCL, VAP and ALH of the two-step group (1:3, 1:2) were significantly higher (*p* < 0.05) than those of the one-step groups (1:5 and 1:11). However, they were not significantly higher (*p* > 0.05) than the two-step group (1:2, 1:1). The post-thawed spermatozoa VSL of the two-step group (1:3, 1:2) was the highest and significantly higher (*p* < 0.05) than that of the two-step group (1:2, 1:1). The post-thawed spermatozoa MAD of the two-step group (1:3, 1:2) was the highest and significantly higher (*p* < 0.05) than that of the one-step group 1:11.

### 3.4. Effect of Different Dilution Methods and Ratios of Diluent on Spermatozoa Membrane Integrity after Cryopreservation

**Figure 1:**
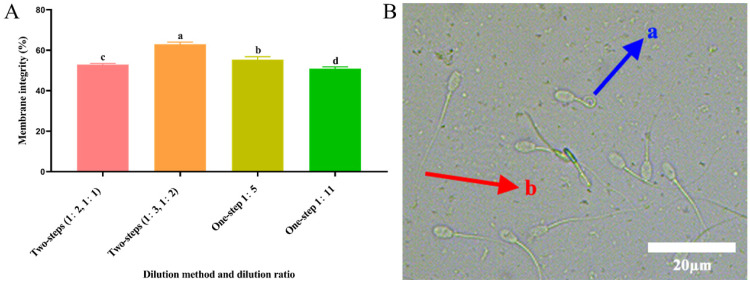
Assessment of plasma membrane integrity. (**A**) Effect of different dilution methods and ratios of diluent on spermatozoa membrane integrity after cryopreservation. Different lowercase letters show significant differences (*p* < 0.05). (**B**) Microscopic results of sperm in HOST experiment. (a) Tail curl represents intact membrane and (b) tail non-curl represents damaged membrane.

As shown in Figure 1A, the post-thawed spermatozoa plasma membrane integrity of the two-step group (1:3, 1:2) was significantly higher (*p* < 0.05) than that of the other groups. As shown in Figure 1B, the blue arrow (a) represents an intact spermatozoa membrane, and the red arrow (b) represents a damaged spermatozoa membrane.

### 3.5. Effect of Different Dilution Methods and Ratios of Diluent on Spermatozoa Acrosome Integrity after Cryopreservation

**Figure 2:**
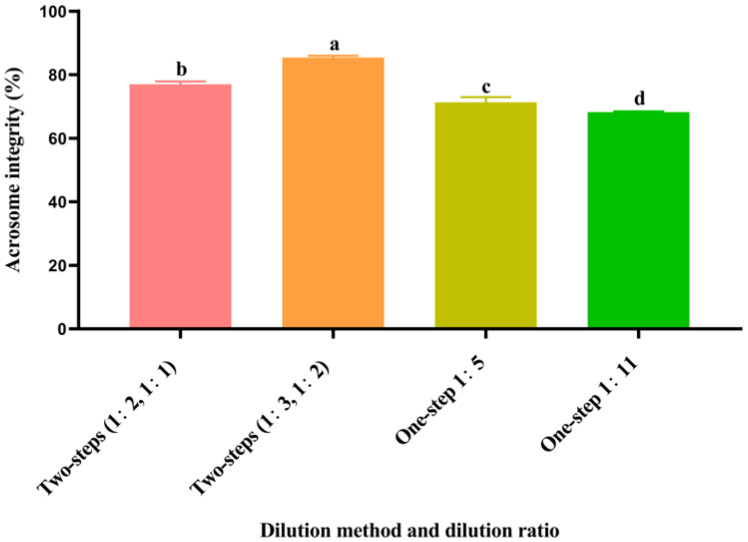
Effect of different dilution methods and ratios of diluent on spermatozoa acrosome integrity after cryopreservation. Different lowercase letters show significant differences (*p* < 0.05).

As shown in Figure 2, the post-thawed spermatozoa acrosome integrity of the two-step group (1:3, 1:2) was significantly higher (*p* < 0.05) than that of the other groups. The post-thawed spermatozoa acrosome integrity of the two-step group (1:2, 1:1) was significantly higher (*p* < 0.05) than that of the one-step groups (1:5 and 1:11). The post-thawed spermatozoa acrosome integrity of the one-step group 1:5 was significantly higher (*p* < 0.05) than that of the one-step group 1:11.

### 3.6. Effect of Different Dilution Methods and Ratios of Diluent on Spermatozoa ROS Level after Cryopreservation

**Figure 3:**
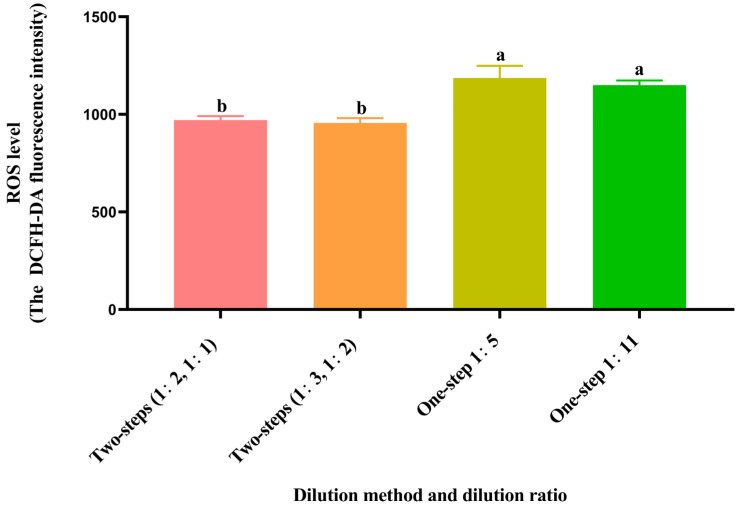
Effect of different dilution methods and ratios of diluent on spermatozoa ROS level after cryopreservation. Different lowercase letters show significant differences (*p* < 0.05).

As shown in Figure 3, the post-thawed spermatozoa ROS levels of the two-step groups (1:2, 1:1 and 1:3, 1:2) were significantly lower (*p* < 0.05) than those of the one-step groups (1:5 and 1:11), but there was no significant difference (*p* > 0.05) in the two-step groups.

## 4. Discussion

Semen dilution simulates the living environment of spermatozoa in vitro. By adding an appropriate diluent, it is possible to reduce the spermatozoa movement rate, inhibit the spermatozoa metabolism, prolong the spermatozoa survival time, increase the semen volume and improve the spermatozoa utilization, thereby increasing the number of breeding female animals [19,20,21]. In experiments I and II, the post-thawed spermatozoa TM and PM of the 1:1 group were significantly lower (*p* < 0.05) than those of the other groups. This may be due to the high concentration of spermatozoa diluted by a factor of two times, the excessive accumulation of metabolic waste and the inability of the buffer material in the diluent to maintain its balance [22]. On the other hand, it may be caused by the rapid consumption of nutrients such as sugar in the diluent [10]. In experiment I, the post-thawed spermatozoa TM and PM of the 1:4 group were significantly lower (*p* < 0.05) than those of the 1:2 group. In experiment II, the post-thawed spermatozoa TM and PM of the 1:4 group were significantly lower (*p* < 0.05) than those of the 1:3 group. This may be due to the fact that a high dilution of semen can cause the glutamic–oxaloacetic transaminase enzyme in spermatozoa to leak out, leading to the death of spermatozoa [23]. This is consistent with Wang’s [24] findings on pig semen, which is highly diluted and causes the leakage of biological enzymes.

In experiment III, the post-thawed spermatozoa in the one-step group 1:11 exhibited significantly lower (*p* < 0.05) plasma membrane integrity and acrosome integrity compared to the other groups. This could be attributed to the reduced antifreeze performance of spermatozoa caused by the higher dilution rate in the one-step dilution method, or it could be due to the absence of essential seminal plasma components when the dilution rate is excessively high [25]. This is consistent with the findings of Wales [26] and Ashworth [27], indicating that high dilution impacts the efficacy of protective components in seminal plasma and the surface structure of the spermatozoa membrane, leading to a reduction in the freezing effect of semen. There is also a saying that the phenomenon is referred to as the dilution effect, which reduces the concentration of natural antioxidants, low-molecular-weight proteins and fatty acids in semen. These substances are beneficial for maintaining the structure and function of the spermatozoa membrane. Excessive dilution of semen can harm the structure of spermatozoa, cause spermatozoa to clump together, significantly shorten the effective preservation time of semen, and subsequently reduce the motility, metabolic activity and fertilization ability of spermatozoa [28,29]. Catt [30] reported that adding seminal plasma had a more positive effect on the cryopreservation of pig semen. Prathalingam [31] reported that the acrosome integrity of bovine semen decreased after thawing when a higher dilution rate was used in cryopreservation. Prochowska [32] reported that the dilution of semen can affect spermatozoa PM and functional integrity in cat semen. Castellini [33] reported that diluting rabbit semen by more than five times will lead to spermatozoa degeneration. These results are consistent with the findings that the integrity of spermatozoa plasma membrane and acrosome decreased as a result of high dilution in this study. The same results were obtained in the changes of spermatozoa membrane integrity and acrosome integrity in the one-step group 1:11. High dilution damages the spermatozoa structure.

In experiment III, the post-thawed spermatozoa TM and PM of the two-step group (1:3, 1:2) were significantly higher (*p* < 0.05) than those of the other groups. Due to the different dilution ratios, the glycerol concentration differs in each diluted group. Therefore, it is difficult to differentiate between the effects of dilution ratios and glycerol concentration in the experiment. On the one hand, this may be due to the fact that a glycerol concentration of 4% in this group is most appropriate for this dilution method and ratio. Proper concentration of glycerol not only enhances the frost resistance of spermatozoa but also prevents the toxic effects of high glycerol concentrations. Glycerol binds to metal ions, dehydrates the cells, reduces the total volume of ice during water solidification, and alleviates the growth of ice crystals, and ultimately reduces the damage caused by freezing [34,35]. On the other hand, this effect may be attributed to the two-step dilution method, which reduces the duration of contact with the spermatozoa and is less harmful when exposed to spermatozoa at low temperatures [36]. This is because the temperature of the diluent is higher when the one-step dilution method is used, which increases the toxic effect of glycerol. In experiment III, the post-thawed spermatozoa ROS level was significantly higher (*p* < 0.05) in the one-step groups (1:5 and 1:11) compared to the other groups. The increase in temperature at the beginning of the process, along with the addition of a glycerol-containing dilution, may have extended the interaction between spermatozoa and glycerol [37]. This prolonged exposure could have intensified the toxic effect of glycerol on spermatozoa, leading to the generation of oxidative stress and an increase in ROS levels.

## 5. Conclusions

In conclusion, the two-step dilution (1:3, 1:2) was found to be the most suitable method and ratio for diluting *Hu* ram semen after cryopreservation. Preserving semen using this dilution method and ratio can effectively enhance the motility parameters and functional integrity of spermatozoa. An excessively high or low spermatozoa concentration will not be conducive to the preservation of *Hu* ram semen.

## Figures and Tables

**Table 1:** Effect of different dilution ratios of diluent I under the condition of 1:1 dilution of diluent II on spermatozoa motility and kinetic parameters after cryopreservation.

Different Dilution Ratios of Diluent I	TM (%)	PM (%)	VSL (µm/s)	VCL (µm/s)	VAP (µm/s)	ALH (µm)	MAD (°/s)
1:1	50.33 ± 1.44 ^c^	31.11 ± 0.99 ^c^	29.28 ± 1.96 ^b^	48.07 ± 2.78	33.99 ± 1.97	14.08 ± 0.82	27.96 ± 0.80 ^c^
1:2	76.86 ± 1.29 ^a^	55.16 ± 0.87 ^a^	34.11 ± 0.59 ^a^	53.03 ± 0.78	37.50 ± 0.55	15.53 ± 0.23	55.71 ± 0.12 ^a^
1:3	71.44 ± 1.57 ^ab^	52.67 ± 0.43 ^ab^	32.68 ± 1.62 ^ab^	50.46 ± 1.50	35.68 ± 1.06	14.78 ± 0.44	51.67 ± 3.36 ^ab^
1:4	70.13 ± 2.25 ^b^	49.65 ± 1.89 ^b^	31.97 ± 0.53 ^ab^	47.88 ± 0.88	33.86 ± 0.62	14.02 ± 0.26	47.76 ± 1.02 ^b^

TM, total motility; PM, progressive motility; VSL, straight-line velocity; VCL, curvilinear velocity; VAP, average path velocity; ALH, amplitude of lateral head displacement; MAD, average motion degree. Different lowercase letter superscripts in the same column show significant differences (*p* < 0.05).

**Table 2:** Effect of different dilution ratios of diluent I under the condition of 1:2 dilution of diluent II on spermatozoa motility and kinetic parameters after cryopreservation.

Different Dilution Ratios of Diluent I	TM (%)	PM (%)	VSL (µm/s)	VCL (µm/s)	VAP (µm/s)	ALH (µm)	MAD (°/s)
1:1	69.51 ± 0.74 ^c^	49.10 ± 0.52 ^c^	33.43 ± 2.14	55.04 ± 1.59 ^ab^	38.92 ± 1.12 ^ab^	16.12 ± 0.46 ^ab^	40.91 ± 3.21 ^b^
1:2	73.45 ± 0.39 ^b^	56.08 ± 0.53 ^b^	32.89 ± 0.53	52.50 ± 1.40 ^b^	37.12 ± 0.99 ^b^	15.38 ± 0.41 ^b^	51.03 ± 2.48 ^ab^
1:3	79.86 ± 0.92 ^a^	63.44 ± 1.55 ^a^	36.54 ± 1.38	58.06 ± 1.40 ^a^	41.05 ± 0.99 ^a^	17.00 ± 0.41 ^a^	58.89 ± 5.59 ^a^
1:4	71.74 ± 1.49 ^bc^	56.50 ± 1.16 ^b^	35.57 ± 0.44	55.77 ± 0.88 ^ab^	39.44 ± 0.62 ^ab^	16.34 ± 0.26 ^ab^	52.97 ± 3.02 ^ab^

TM, total motility; PM, progressive motility; VSL, straight-line velocity; VCL, curvilinear velocity; VAP, average path velocity; ALH, amplitude of lateral head displacement; MAD, average motion degree. Different lowercase letter superscripts in the same column show significant differences (*p* < 0.05).

**Table 3:** Effect of different dilution methods and ratios of diluent on spermatozoa motility and kinetic parameters after cryopreservation.

Different Dilution Methods and Ratios	TM (%)	PM (%)	VSL (µm/s)	VCL (µm/s)	VAP (µm/s)	ALH (µm)	MAD (°/s)
Two-step: 1:2, 1:1	77.01 ± 2.07 ^b^	60.83 ± 1.89 ^b^	38.08 ± 1.23 ^b^	57.68 ± 1.84 ^ab^	40.78 ± 1.30 ^ab^	16.89 ± 0.54 ^ab^	49.19 ± 4.98 ^ab^
Two-step: 1:3, 1:2	85.44 ± 2.07 ^a^	72.68 ± 0.59 ^a^	42.28 ± 0.34 ^a^	62.41 ± 0.66 ^a^	44.13 ± 0.46 ^a^	18.28 ± 0.19 ^a^	53.90 ± 1.46 ^a^
One-step: 1:5	78.83 ± 1.24 ^b^	60.57 ± 1.40 ^b^	40.14 ± 1.15 ^ab^	56.34 ± 2.33 ^b^	39.83 ± 1.65 ^b^	16.50 ± 0.68 ^b^	42.38 ± 1.73 ^ab^
One-step: 1:11	76.83 ± 1.71 ^b^	60.09 ± 1.01 ^b^	40.79 ± 0.91 ^ab^	55.47 ± 1.44 ^b^	39.22 ± 1.02 ^b^	16.24 ± 0.42 ^b^	39.07 ± 5.86 ^b^

TM, total motility; PM, progressive motility; VSL, straight-line velocity; VCL, curvilinear velocity; VAP, average path velocity; ALH, amplitude of lateral head displacement; MAD, average motion degree. Different lowercase letter superscripts in the same column show significant differences (*p* < 0.05).

## Data Availability

All data sets collected and analyzed during the current study are available from the corresponding author (Y.L.) upon reasonable request.
